# Novel candidate genes for cholesteatoma in chronic otitis media

**DOI:** 10.3389/fgene.2022.1033965

**Published:** 2023-01-09

**Authors:** Nam K. Lee, Stephen P. Cass, Samuel P. Gubbels, Helen Z. Gomez, Melissa A. Scholes, Herman A. Jenkins, Regie Lyn P. Santos-Cortez

**Affiliations:** ^1^ Department of Otolaryngology—Head and Neck Surgery, School of Medicine, University of Colorado Anschutz Medical Campus, Aurora, CO, United States; ^2^ Human Medical Genetics and Genomics Program, University of Colorado Anschutz Medical Campus, Aurora, CO, United States; ^3^ Department of Pediatric Otolaryngology, Children’s Hospital Colorado, Aurora, CO, United States; ^4^ Center for Children’s Surgery, Children’s Hospital Colorado, Aurora, CO, United States

**Keywords:** cholesteatoma, otitis media, exome sequencing, network analysis, chronic otitis media

## Abstract

Cholesteatoma is a rare and benign disease, but its propensity to cause erosive damage through uninhibited growth can be detrimental to hearing and health. Prior reports indicated a genetic component to pathogenesis in at least a subset of patients. In this study, we aimed to identify rare DNA variants in affected patients. The salivary DNA of six patients whose middle ear tissues were obtained during tympanoplasty/mastoidectomy surgeries were submitted for exome sequencing. Tissue samples from the same patients were previously submitted for mRNA sequencing and analyzed for differentially expressed genes (DEGs). From the generated exome sequence data, rare predicted-to-be-damaging variants were selected within previously identified DEGs, and the candidate genes within which these rare variants lie were used for network analysis. Exome sequencing of six DNA samples yielded 5,078 rare variants with minor allele frequency <.001. A total of 510 variants were predicted to be deleterious and 52 were found to lie within previously identified DEGs. After selecting variants based on quality control measures, 12 variants were identified all from one pediatric patient. Network analysis identified ten significant cellular pathways, including protein transport, viral process, regulation of catalytic activity and cell cycle, and apoptotic and rhythmic processes. We hypothesize that the candidate genes identified in this study may be part of key signaling pathways during the mucosal response to middle ear infection. The occurrence of multiple rare variants may play a role in earlier onset of cholesteatoma formation in chronic otitis media.

## 1 Introduction

Cholesteatoma is a rare and benign disease of the middle ear, affecting approximately one per 10,000 individuals, but due to its potential for growth, may result in a wide array of detrimental consequences (Kemppainen et al., 1999). This cyst-like mass containing keratinous debris with patchy squamous epithelial lining can grow beyond the boundaries of the middle ear leading to erosion of the temporal bone. Conductive and sensorineural hearing loss, soft tissue abscesses, facial paralysis, and meningitis, among other potential complications, may follow the destruction of the middle and inner ear, facial nerve, or skull base (Kuo et al., 2015). Existing treatment options lack any medical alternatives and are limited to surgery. The surgical treatment of cholesteatoma is often conducted in multiple stages and requires careful and frequent follow-up to assess for any recurrence or regrowth of residual disease (Kuo et al., 2015).

There are two distinct categories of cholesteatoma: Congenital, found behind an intact tympanic membrane, and acquired, often accompanying chronic infection of the middle ear. Between its two forms, acquired cholesteatoma presents more frequently with over 70% of cases in association with a history of chronic otitis media (Kemppainen et al., 1999). With an understanding of chronic infection and inflammation as the basis for disease origin, numerous theories on the pathogenesis of cholesteatoma were developed (Castle, 2018). Although emerging evidence implicates contrasting theories, syndromic and familial forms of cholesteatoma suggest a role of genetics in the disease process, thus providing an alternative approach and credence to further investigations into the genetic basis for cholesteatoma formation (Bacciu et al., 2005; Prinsley, 2009; Lim et al., 2014; Jennings et al., 2018; Collins et al., 2020).

Genomic investigation can uncover key insights into rare diseases through the identification of rare but high-penetrance variants with complex inheritance patterns. Cholesteatoma has been associated with syndromes with chromosomal abnormalities or genetic variants, such as Turner, Down, and Branchio-oto-renal syndromes (Lipkin et al., 1986; Bacciu et al., 2005; Bergamaschi et al., 2008; Vaglio et al., 2008; Lim et al., 2014; Diego et al., 2018). Familial patterns of cholesteatoma have also been described in siblings, twins, and intergenerational families. In a recent study of 857 individuals, 10.4% reported a family history of cholesteatoma, which in turn was a risk factor for bilateral disease (OR 2.15; 95% CI: 1.35, 3.43; *p* = .001) (Collins et al., 2020). In a follow-up study by Prinsley et al. (2019), who identified three familial cases of cholesteatoma in a rural region of the United Kingdom, two rare loss-of-function variants in *EGFL8* and *BTNL9* were identified in one of the families (Prinsley, 2009). Based on these associations and patterns of disease presentation, there is the potential to identify genetic drivers of cholesteatoma pathogenesis and pathways that are potential targets for effective medical solutions.

In a previous study that profiled the expression of cholesteatoma, differentially expressed genes (DEGs) were identified using three cholesteatoma and three middle ear mucosal samples of six unrelated patients with cholesteatoma (Baschal et al., 2020). Contrarily, in this report, DNA extracted from saliva samples of the same six patients with cholesteatoma were submitted for exome sequencing to identify rare variants that are predicted to be damaging. We hypothesized that at least some of the DEGs identified in our previous mRNA-seq study were potentially due to rare genetic variants in the patients who provided both mRNA-seq and exome data. Identified candidate genes were then submitted for network analysis to identify protein-protein interactions and cellular pathways that are potentially involved in cholesteatoma pathogenesis.

## 2 Materials and methods

### 2.1 Ethical approval, recruitment, and sample collection

The study was approved by the Colorado Multiple Institutional Review Board (protocol 16-2673) prior to initiation. Informed consent was obtained from all study participants. The relevant clinical data from these patients were collected using the Research Electronic Data Capture (REDCap), a HIPAA-compliant electronic data capture tool (Supplementary Table S1).

In a prior study, cholesteatoma or middle ear mucosal tissues were collected in six unrelated patients with cholesteatoma (Baschal et al., 2020). From the three cholesteatoma and three middle ear mucosal tissues of the six patients, RNA was isolated and submitted for bulk mRNA-sequencing. Then, using the resulting mRNA-seq data, 1,806 DEGs were identified for cholesteatoma.

For this exome filtering study, saliva samples of the same six patients with mRNA-seq and DEG data were collected using Oragene OGR-500 kits (DNAgenotek, Ottawa, Ontario, Canada) and used to extract DNA per the manufacturer’s protocol.

### 2.2 Exome sequencing and analysis

The DNA extracted from six saliva samples were submitted for exome sequencing at the University of Washington Northwest Genomics Center. The Twist Bioscience Human Core Exome Kit (South San Francisco, CA, United States of America) was used for sequence capture, and sequencing was performed using an Illumina HiSeq (San Diego, CA, United States of America) to an average depth of 30×. Fastq files were aligned to the hg38 human reference sequence using Burrows-Wheeler Aligner, generating demultiplexed .bam files (Li and Durbin, 2009). The Genome Analysis Tool Kit was used for variant detection and calling, as well as generation of standard metrics used for quality control (QC) during exome analyses (McKenna et al., 2010). Low-quality and likely false-positive variants were flagged. The generated .vcf file was annotated using ANNOVAR (Wang et al., 2010).

Annotated variants were selected based on the following criteria: 1) minor allele frequency (MAF) ≤.001 in any known population in public genome databases including gnomAD, 1,000 Genomes, or Greater Middle East (GME) Variome (Lek et al., 2016; Scott et al., 2016; Azaiez et al., 2018); 2) scaled Combined Annotation Dependent Depletion score of ≥3 and/or had dbscSNV score ≥.7 (Kircher et al., 2014); (3a) predicted to be deleterious by at least one bioinformatics tool included in dbSNFP33a in the case of missense or stop variants (Liu et al., 2011; Liu et al., 2013; Liu et al., 2016); or (3b) predicted to be deleterious by MutationTaster for splice and frameshift variants (Steinhaus et al., 2021); 4) lie within DEGs that were previously identified using middle ear samples from the same six patients with exome data (Baschal et al., 2020); and 5) pass QC filters. In addition to the dbscSNV score, missense variants were also rechecked against equivalent hg19 databases then annotated using MutationTaster (Kent et al., 2002; Steinhaus et al., 2021). For the final set of candidate genes with rare deleterious variants, MAF was also checked in the GenomeAsia 100K database and variants were visualized from .bam files using the Integrative Genomics Viewer (IGV) v2.12.3 (Thorvaldsdóttir et al., 2013; GenomeAsia100K Consortium, 2019). For further validation, Sanger sequencing of the identified variants was performed using the DNA sample from one pediatric patient.

### 2.3 Network analysis

NetworkAnalyst software and the IMEx Interactome database were used to visualize protein-protein networks with the candidate genes as input (Xia et al., 2014; Xia et al., 2015; Zhou et al., 2019). For the resulting subnetworks, enrichment analysis was conducted using PANTHER BP (Mi et al., 2019). False discovery rate (FDR)-adjusted *p* < .05 was used as the threshold to denote significant pathways.

## 3 Results

### 3.1 Exome analysis

Among the six patients with cholesteatoma who provided both DNA and RNA samples, the average age was 42 years (range 9.6–77.3 years) and all patients were male (Baschal et al., 2020). Five patients identified as White and one patient as Hispanic. Clinical description of patients including otoscopy, imaging, and intra-operative findings can be found in Supplementary Table S1.

**FIGURE 1:**
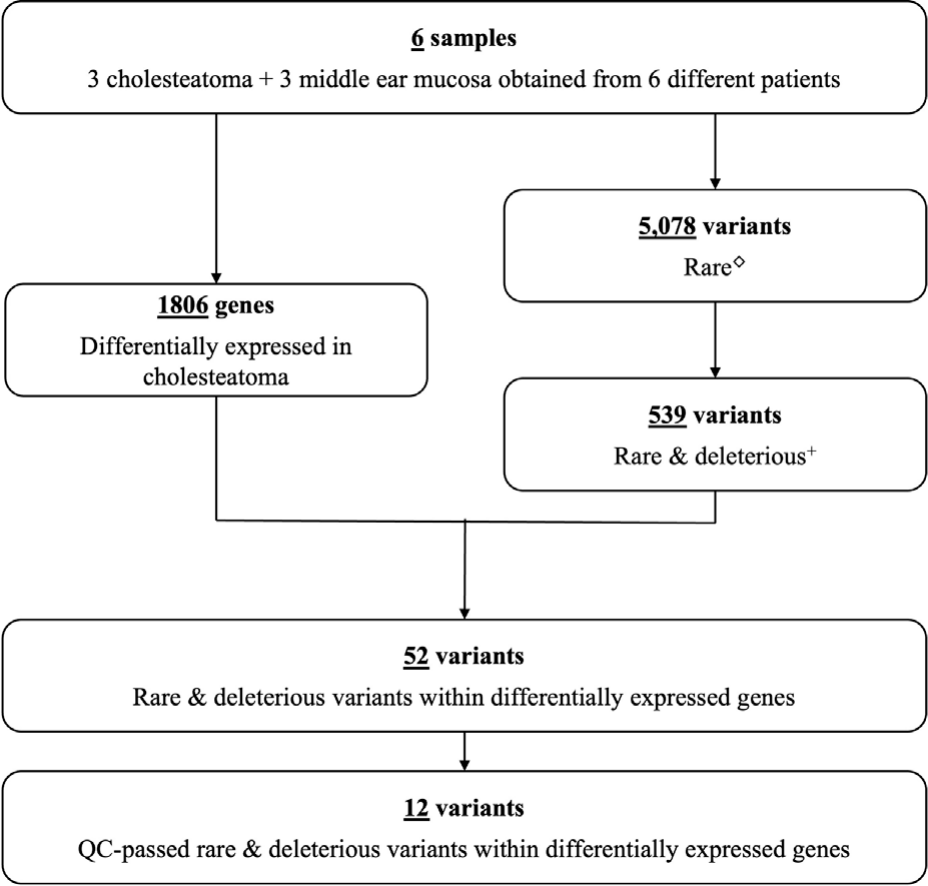
Study flow chart. Number of differentially expressed genes and genetic variants identified in six patients with cholesteatoma (Supplementary Table S1). ^◇^Rare variants with MAF ≤.001. ^+^Predicted to lead to loss of gene function.

From the combined data of six patients, exome sequencing revealed a total of 113, 176 genetic variants within the coding regions of the human genome. Of these variants, 5,078 were rare with a MAF ≤.001 (Figure 1). Five-hundred thirty-nine of these rare variants had a scaled CADD score ≥3. Of these, 426 were single nucleotide variants (SNVs) predicted to be deleterious by at least one bioinformatics tool, and 41 of these SNVs lie within our previously identified DEGs for cholesteatoma (Baschal et al., 2020). On the other hand, there were 113 splice or frameshift variants, of which 84 were predicted to be deleterious, and 12 frameshift variants were found within DEGs. In total, 52 rare deleterious variants were found within DEGs and 12 passed QC filters. In summary, 12 rare deleterious variants, including one frameshift and 11 missense variants, were included in the final selection (Table 1).

**TABLE 1:** Twelve candidate genes identified in a pediatric patient with cholesteatoma.

Gene	Variant [rsID from dbSNP]	gnomAD^a^	CADD	Damaging predictions	mRNA-Seq levels Baschal et al. (2020)				
					Patient	Chol avg	Muc avg	*p*-value	FDR-adj-*p*
*RTN4*	NM_207520:c.902G>A [p.(Cys301Tyr)]	NA	24	PP2_HDIV, PP2_HVAR, MT, MA, PROVEAN	1202.5	863.2	352.2	.002	.02
*RAB5A*	NM_001292048:c.481A>G [p.(Met161Val)]	AFR:6.2 × 10^−5^	19.5	LRT, MT, MA, mRNN, MCAP	323.7	273.0	110.4	.003	.03
*CRYBG1*	NM_001371242:c.1366G>A [p.(Ala456Thr)]	AMR:0.0002	23.4	SIFT, PP2_HDIV	1927.3	1654.5	603.6	.001	.02
*RGS22*	NM_001286692:c.19A>G [p.(Thr7Ala)] [rs993516236]	SAS:5.49 × 10^−5^	13.9	MA	0.3	1.5	0.3	5.6 × 10^−4^	.01
*APBB1IP*	NM_019043:c.535G>A [p.(Val179Ile)] [rs750180116]	SAS:0.0001; AMR:2.89 × 10^−5^; NFE:8.80 × 10^−6^; OTH:0.0002	22.6	PP2_HDIV, PP2_HVAR, LRT, MT, MA	5.6	8.3	55.5	.003	.03
*HEPHL1*	NM_001098672:c.1123A>G [p.(Met375Val)] [rs756695159]	AMR:0.0006	8.8	FATHMM, mSVM, mLR, MCAP	56.4	22.0	0	.002	.03
*BHLHE41*	NM_030762:c.373C>G [p.(Gln125Glu)] [rs371168594]	AMR:0.0003; NFE: 9.27 × 10^−6^; OTH:0.0002	22	MT, MA	6.9	14.0	117.4	.0001	.003
*ARID3A*	NM_005224:c.553G>A [p.(Gly185Ser)] [rs911982273]	AMR:0.0003	9.5	PP2_HDIV, MCAP	9.9	11.6	94.2	.001	.02
*C5AR1*	NM_001736:c.491C>A [p.(Ala164Asp)] [rs145736934]	AFR:0.001	23.5	SIFT, SIFT4G, PP2_HDIV, PP2_HVAR, MT, PROVEAN, mSVM, mRNN, MCAP, REVEL	24.6	24.3	179.7	.0008	.01
*SPTLC3*	NM_001349945:c.188T>C [p.(Met63Thr)] [rs749277943]	SAS:3.3 × 10^−5^; AMR:0.0001; NFE:8.9 × 10^−6^	20.5	SIFT, SIFT4G	845.3	850.0	173.6	3.7 × 10^−6^	.0003
*CPT1B*	NM_001145134:c.239C>T [p.(Ser80Phe)] [rs745528078]	AMR:0.0007; OTH:0.0003	27.4	SIFT, PP2_HDIV, PP2_HVAR, LRT, MT, FATHMM, PROVEAN, mLR, mSVM, mRNN, MCAP, REVEL	11.9	18.5	58.9	.004	.04
*FAM227A*	NM_001013647:c.637_646del [p.(Gln213Cysfs*5)]	AMR:0.0002	NA	MT	2.1	2.2	104.7	1.1 × 10^−4^	.003

^a^ Highest gnomAD MAF estimates are reported.

Abbreviations: gnomAD, genome aggregation database v3.1.1; Chol avg, average of normalized reads for OM cholesteatoma samples; Muc avg, average of normalized reads for middle ear mucosa samples; FDR-adj-*p*, false discovery rate adjusted *p*-value using the Benjamini–Hochberg method; CADD, combined annotation-dependent depletion score; PP2_HDIV, PolyPhen2 HDIV database; PP2_HVAR, PolyPhen2 HVAR database; MT, Mutation taster; MA, mutation assessor; PROVEAN, protein variation effect analyzer; LRT, likelihood ratio test; MRNN, MetaRNN; MCAP, Mendelian clinically applicable pathogenicity; SIFT, sorting intolerant from tolerant; SIFT4G, SIFT for genomes; FATHMM, functional analysis through hidden Markov models; mLR, MetaLR; mSVM, MetaSVM; REVEL, rare exome variant ensemble learner.

The 12 selected variants were identified as heterozygous in one pediatric patient and were verified by Sanger sequencing; however, no rare variants were identified in the other five adult patients (Table 1). These 12 variants were found in the following genes: *APBB1IP, ARID3A, BHLHE41, C5AR1, CPT1B, CRYBG1, FAM227A, HEPHL1, RAB5A, RGS22, RTN4,* and *SPTLC3*. All 12 genes are novel and have not been previously reported for DNA variants that confer human susceptibility to otitis media or cholesteatoma.

### 3.2 Network analysis

**FIGURE 2:**
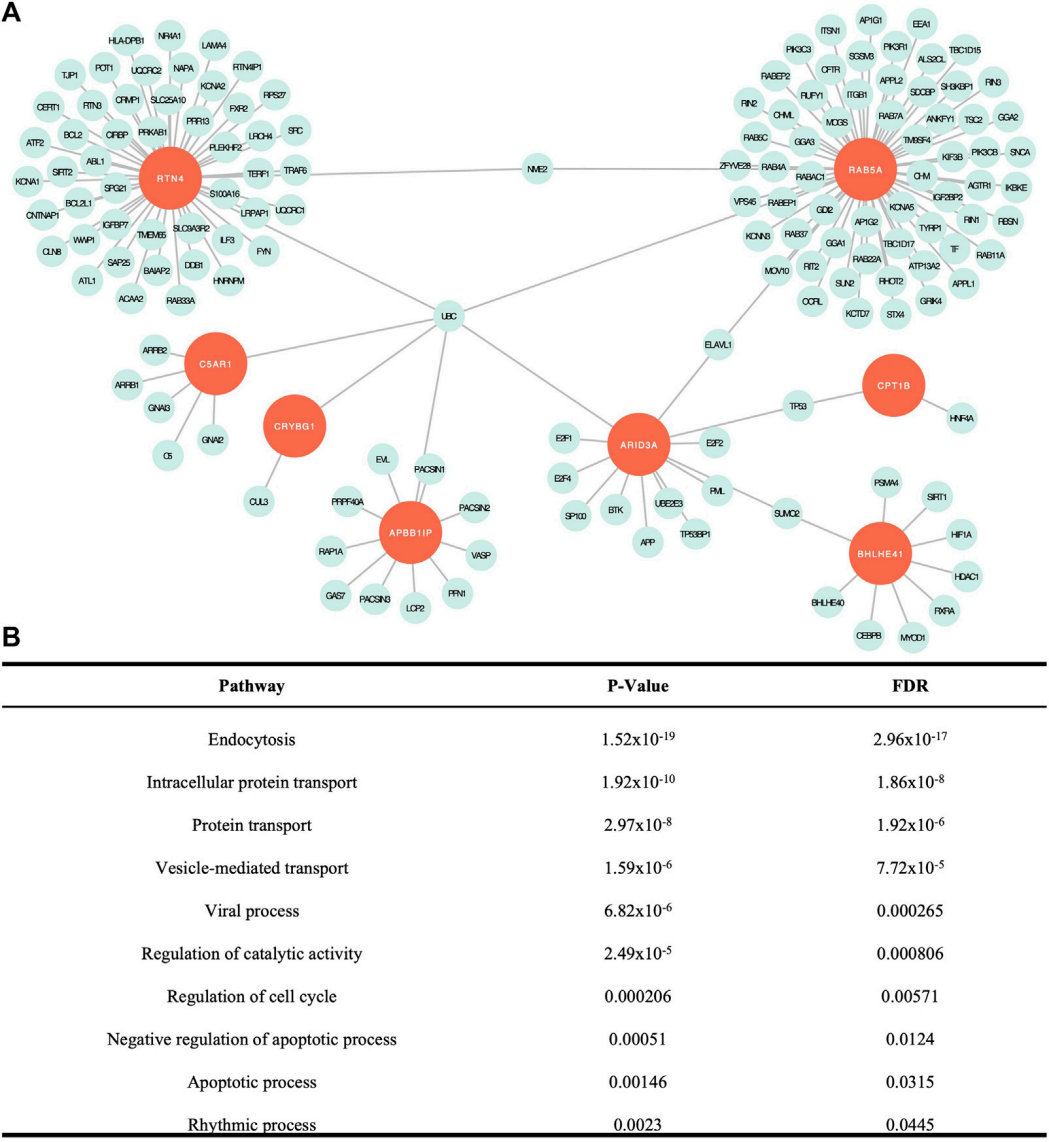
Network analysis findings. **(A)** Visual representation of the network created with the eight seed genes and associated proteins. **(B)** Significant cellular pathways determined by false discovery rate (FDR) adjusted *p* < .05. All except intracellular protein transport overlapped with significant pathways identified from DEG data (Baschal et al., 2020).

Network analysis is used to build a network of genes that interact to serve a set of biological functions. In this model, seeds are the genes from the list of input genes, in this case, our 12 candidate genes, which interact within the biological pathways identified in the network. Nodes represent genes that work collectively to serve a biological process and edges represent interactions between the nodes. Our analysis yielded a subnetwork consisting of 157 edges and 156 nodes (Figure 2A). Of the 12 candidate genes, eight genes were included as seeds in the network, including *APBB1IP, ARID3A, BHLHE41, C5AR1, CPT1B, CRYBG1, RAB5A,* and *RTN4*. There were 23 cellular pathways identified within the subnetwork, 10 of which were significant (Figure 2B). A PANTHER BP pathway was deemed significant if the module analysis on each subnetwork led to an FDR-adjusted *p*-value <.05. The cellular and biological functions were classified using PANTHER BP Gene Ontology Terms. The significant pathways identified in our network analysis included endocytosis, intracellular protein transport, protein transport, vesicle-mediated transport, viral process, regulation of catalytic activity, regulation of cell cycle, negative regulation of apoptotic process, apoptotic process, and rhythmic process. When compared to the network analysis conducted using DEGs from tissue mRNA-sequence data as input, 17 cellular processes out of the 23 identified in our study overlapped (Supplementary Table S2).

## 4 Discussion

**FIGURE 3:**
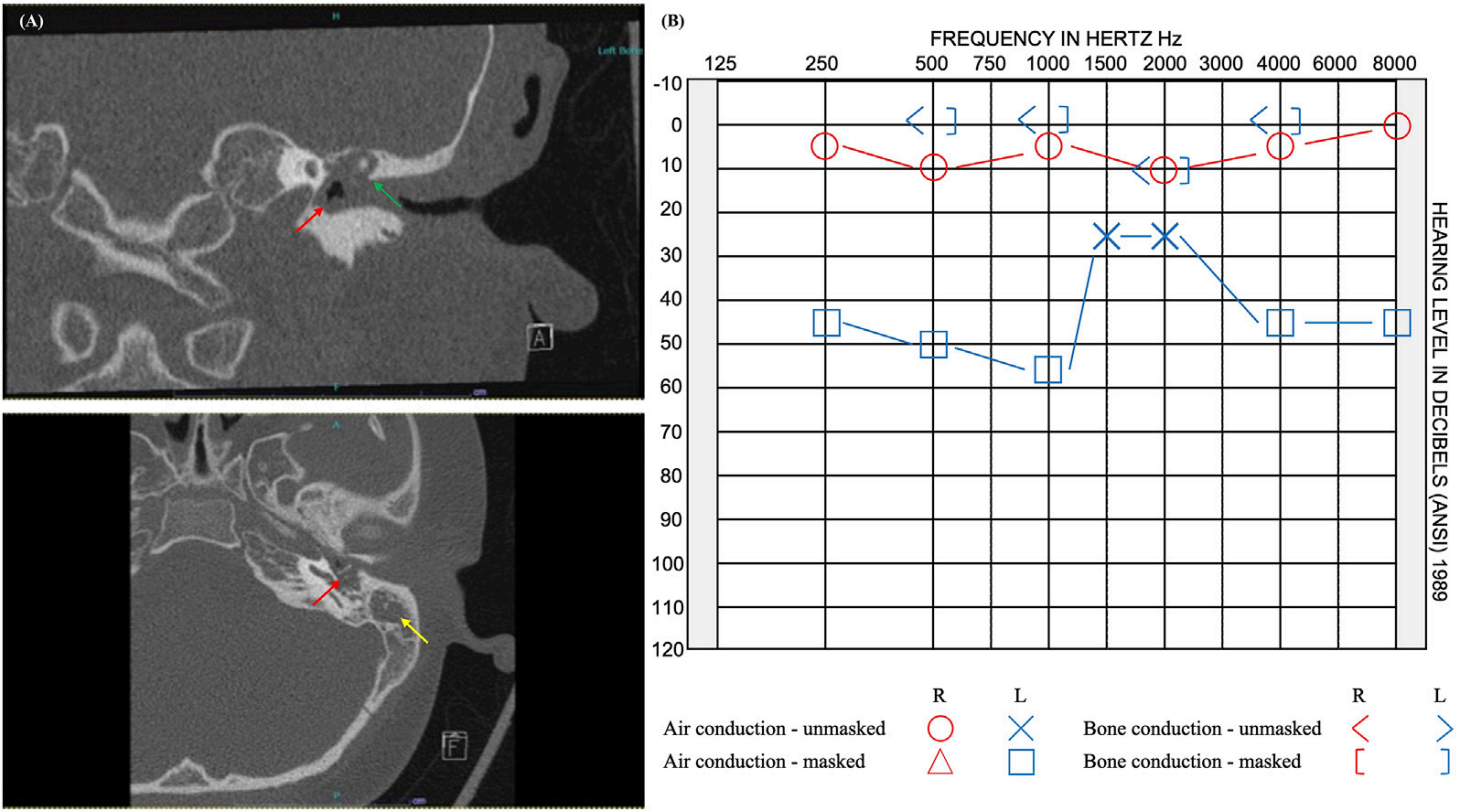
Imaging and audiogram results of pediatric patient with rare variants in 12 candidate genes. **(A)** CT imaging reveals opacification of the left middle ear cavity (red arrow), blunting of the scutum (green arrow), and complete opacification of the left mastoid (yellow arrow). **(B)** Audiogram results showed normal hearing at 250–8,000 Hz on the right and moderate rising to mild conductive hearing loss at 250–8,000 Hz on the left.

In this study, we report 12 novel candidate genes for cholesteatoma that were identified from the exome sequence data while using DEGs from a prior mRNA-sequencing study of the same six unrelated patients (Baschal et al., 2020). All the 12 candidate genes were identified in the youngest of the cohort, who presented with cholesteatoma at 9 years old (Figure 3). The patient’s cholesteatoma is consistent with the acquired form that started with an episode of acute otitis media long before the diagnosis of right-sided conductive hearing loss at age 8 years. His otoscopic examination revealed a white mass behind a retracted left tympanic membrane and moderate conductive hearing loss in the same ear. Although the extent of his disease was limited to the middle ear and less severe than the other individuals in the study, the relatively early presentation of cholesteatoma in the context of an indistinct history of acute otitis media is notable. While acquired cholesteatoma is usually observed in patients with chronic otitis media, there have been previous reports of cholesteatoma diagnosed following an episode of acute otitis media, that is potentially infected by atypical bacteria (Abes et al., 2017). However, in our patient, it is more likely that there was inadequate documentation of chronic otitis media.

The expression profiles of the 12 candidate genes were assessed in the online databases, Genotype-Tissue Expression (GTEx) and Human Protein Atlas (Lonsdale et al., 2013; Uhlén et al., 2015). Due to the absence of middle ear tissues in these databases, esophageal mucosa, lungs, cervix, uterus, vagina, minor salivary glands, and skin were assessed as surrogates for middle ear mucosa (Supplementary Table S2). All 12 genes showed expression at the RNA or protein level in the tissues of interest (Supplementary Table S2). None of the genes had prior implications in OM or other middle ear pathologies to the best of our knowledge. However, in our own middle ear expression dataset from the same patients, all 12 genes were DEGs for cholesteatoma (Table 1): *RTN4, RAB5A, CRYBG1, RGS22, HEPHL1,* and *SPTLC3* were upregulated, while *APBB1IP, BHLHE41, ARID3A, C5AR1, CPT1B,* and *FAM227A* were downregulated. The pediatric patient with variants in all 12 genes had similar trends in expression, except for *RGS22,* which had reduced expression with levels similar to the mucosal samples than to the other cholesteatoma samples (Table 1).

From the 12 candidate genes, eight were identified as seeds in our network analysis: *APBB1IP, ARID3A, BHLHE41, C5AR1, CPT1B, CRYBG1, RAB5A,* and *RTN4.* These seed genes all play a role in the innate immune response to infection or disease (Supplementary Table S3). The innate immune system has been historically implicated in the pathogenesis of otitis media (Ram and Chinen, 2011). A recent single-cell transcriptomic study of a normal murine middle ear illustrated the expression of innate immunity-related genes in all 17 mucosal cell types (Ryan et al., 2020).

Major cellular pathways in our network analysis relate to endoplasmic reticulum (ER) function, including intracellular protein transport, protein transport, and vesicle-mediated transport. When otitis media is induced, a rise in ER stress function is observed with an associated increase in proinflammatory gene expression (Zhao et al., 2020). ER dysfunction is known to cause various inflammatory diseases (Cao et al., 2013; Zhang et al., 2017). The additional basic cellular processes highlighted in our study include poor wound healing and cell renewal in cholesteatoma. While viral process was a significant pathway, our previous study on the microbiota of cholesteatoma did not detect any of the common respiratory viruses (Frank et al., 2022). Taken together, the 12 candidate genes may be part of key signaling pathways during the mucosal response to a middle ear infection.

There are several limitations to this study. Due to the rarity of cholesteatoma, we have a very limited sample size with a methodology involving rare variant detection from exome sequence data. Note, however, that there has not been any previous genome-wide association studies published on a cholesteatoma cohort to identify significant genes (Baschal et al., 2020). Validation of the variants identified in this study may be best achieved using functional analysis with mutations induced in the 12 identified variants. The current lack of a clear understanding of the potential tissue source or cell type that evolves into cholesteatomatous tissue limits the design of functional experiments in a suitable cell line. On the other hand, our review of the literature demonstrated mouse model, cell line, or disease entities with the same direction of effect as in cholesteatoma samples in 7 of the 12 candidate genes (e.g., gene knockout vs. downregulation in cholesteatoma) with immune cell defects affecting the innate immune defenses (Supplementary Table S3).

## 5 Conclusion

We identified 12 novel candidate genes for cholesteatoma supported by identification of both genetic variants from exome sequence data and DEGs from mRNA-seq using cholesteatoma and middle ear mucosal tissues. Out of the 12 rare coding variants identified in a single Hispanic-American child with cholesteatoma, 11 were found as heterozygous in various gnomAD populations (Table 1), indicating that these variants may be replicated in DNA sequencing studies of cholesteatoma patients from other world populations. Our findings might also suggest that a few of the DEGs identified in cholesteatoma have a genetic basis, rather than being a product of chronic infectious processes. The occurrence of multiple rare variants might also play a role in earlier onset of cholesteatoma formation in chronic otitis media. Identification of these genes and pathways not only enriches our understanding of cholesteatoma formation but improve the potential for developing new prevention and treatment strategies for otitis media.

## Data Availability

The datasets presented in this study can be found in online repositories. The names of the repository/repositories and accession number(s) can be found below: https://www.ncbi.nlm.nih.gov/gap/, phs001941 v1.p1.
