# Supplementary material for: Novel candidate genes for cholesteatoma in chronic otitis media
Source: Front Genet. 2023 Jan 9;13:1033965. doi: 10.3389/fgene.2022.1033965 (PMC9868167; doi:10.3389/fgene.2022.1033965)
Supplement: Supplementary file 1 [file DataSheet1.docx]

| **Supplementary Table 1. Clinical profiles of patients with cholesteatoma included in this study** | | | | | | |
| --- | --- | --- | --- | --- | --- | --- |
| **ID** | **Tissue**  **Type** | **Ethnicity** | **Age**  **(yrs)** | **Otoscopic Findings** | **CT Findings** | **Intraoperative Findings** |
| **1** | Chol | Hispanic | 9.6 | Left TM is retracted, whitish mass behind eardrum, malleus remnant visualized | - Near complete opacification of left EAC and middle ear cavity - Complete opacification of left mastoid - Blunting of the scutum and demineralized appearance of the ossicles suggests underlying cholesteatoma | - Chorda tympani preserved but stretched - Ossicular chain intact and mobile - Anterior TM atelectatic to promontory, posterior TM retraction pocket posteriorly and into sinus tympani with small cholesteatoma in posterior mesotympanum and sinus tympani. No epitympanic or mastoid extent, however likely not aerated due to atelectatic drum and retraction pocket. Mastoidectomy not indicated |
| **2** | Mucosa | White | 19.3 | - Right TM is intact with grafted cartilage - Left with posterior mesotympanic retraction pocket (Sade grade 4) | NA | - Recurrent pearl of cholesteatoma underneath the neck of the malleus, going up to the epitympanum. - No facial nerve abnormalities, dehiscence, stimulation, or aberrant course.  Chorda tympani nerve was not present.  Incus and malleus were not present |
| **3** | Mucosa | White | 20 | - Right TM is intact with no inflammation, retraction or perforation noted. Right middle ear is normal; no fluid or cholesteatoma noted. - Left TM with attic retraction and crusting. Left middle ear with white mass causing bulging of the TM posteriorly | - Left middle ear mass involving epitympanum and mastoid antrum- compatible with cholesteatoma - No labyrinthine erosion noted | - Extensive middle ear mastoid cholesteatoma. - The cholesteatoma filled the entire middle ear space, at the tympanic spaces, mastoid antrum, and facial recess. - The cholesteatoma extended into the petrosal portion of the temporal bone in the area of the geniculate ganglion and medial to the ampulla of the superior semicircular canal. - Gross total tumor removal was accomplished taking extended operative time. |
| **4** | Chol | White | 61.4 | - Right TM has an area of debris going under the scutum into Prussak's space; otherwise there is normal mobility; no inflammation, retraction or perforation noted. Right middle ear is normal; no fluid or cholesteatoma noted. - Left TM has normal mobility; no inflammation, retraction or perforation noted. Left middle ear is normal; no fluid or cholesteatoma noted. | Cholesteatoma in Prussak's space with no extension into mastoid antrum | - Cholesteatoma present in epitympanum - No erosion of ossicles |
| **5** | Chol | White | 66.4 | - Right TM has normal mobility; no inflammation, has retraction under the scutum noted. Right middle ear is normal; no fluid or cholesteatoma noted. - Left TM has normal mobility; no inflammation, retraction or perforation noted. Left middle ear is normal; no fluid or cholesteatoma noted. | - Right EAC soft tissue alongside retracted and thickened TM with lateral epitympanic soft tissue and associated partial osseous erosions of the scutum and ossicles as above - Findings most suspicious for cholesteatoma | - Left TM is retracted but stable - Keratinaceous debris consistent with cholesteatoma within a deep posterosuperior retraction pocket. There was extension laterally into the EAC deep to the mucosa but without significant bony destruction - Prior cartilage graft involved in cholesteatoma - Malleus, incus interposition graft, and stapes present, but not in continuity. No cholesteatoma involvement of the ossicles |
| **6** | Mucosa | White | 77.3 | - Right TM is intact with no inflammation, retraction or perforation noted. Right middle ear is normal; no fluid or cholesteatoma noted - Left TM has a posterior TM perforation with keratin debris tracking in the undersurface of the TM.  The TM is thicker than normal and opaque so unclear the extent of cholesteatoma. Left middle ear could not be assessed | Soft tissue within the left middle ear with possible erosion of the stapes crura, which could represent granulation tissue or cholesteatoma | - Small posterior superior quadrant perforation with extensive cholesteatosis of the middle ear - Cholesteatoma tracked to involve the undersurface of the entire eardrum as well as to make deposits within the middle ear space.  There was no encapsulated cyst - Ossicular chain was found to be intact - No cholesteatoma found within the mastoid antrum or epitympanum |
| Chol: cholesteatoma; CT: computed tomography; EAC: external auditory canal; TM: tympanic membrane. All six recruited patients are male. | | | | | | |

| **Supplementary Table 2. Pathways from network analysis that overlapped between Baschal et al. 2020 and this study** | |
| --- | --- |
| **Pathway** | **FDR-adj-*p**** |
| Endocytosis | 2.96x10^-17^ |
| Protein transport | 1.92x10^-6^ |
| Vesicle-mediated transport | 7.72x10^-5^ |
| Viral process | 0.0003 |
| Regulation of catalytic activity | 0.0008 |
| Regulation of cell cycle | 0.006 |
| Negative regulation of apoptotic process | 0.01 |
| Apoptotic process | 0.03 |
| Rhythmic process | 0.044 |
| Cell cycle | 0.08 |
| Phagocytosis | 0.09 |
| Cell proliferation | 0.13 |
| Cytoskeleton organization | 0.14 |
| Circadian rhythm | 0.18 |
| Blood coagulation | 0.18 |
| Transcription by RNA polymerase II | 0.18 |
| Protein phosphorylation | 0.34 |
| *These FDR-adjusted p-values are based on module analysis of the subnetwork created when including 8 out of the 12 candidate genes from this study as seeds in NetworkAnalyst. | |

| **Supplementary Table 3. RNA and protein expression profiles in various human tissues** | | | |
| --- | --- | --- | --- |
| **Gene** | **Tissue with highest RNA expression^1^**  GTEx Consortium | **Protein expression**  Human Protein Atlas | **Function**  PubMed |
| *APBB1IP* | Whole blood, EBV-transformed lymphocytes, spleen, adipose tissues | Cerebellum, testis, appendix, spleen, lymph node | In hematopoietic cells, involved in phagocytosis, including particle recognition, cytoskeletal remodeling and membrane protrusion for engulfment and digestion (Sari-Ak et al., 2022). Knockout mice have IL-10 deficiency but are protected from autoimmune colitis and have preserved Treg cell trafficking and function (Sun et al., 2021) |
| *ARID3A* | EBV-transformed lymphocytes, testis, whole blood, cultured fibroblasts, spleen | Placenta, testis, tonsil, cerebral cortex, thyroid gland | TLR signaling induces *ARID3A* expression in hematopoietic progenitors in association with Type I interferon inflammatory cytokines (Ratliff et al., 2020). Both transcription factors *ARID3A* and *BHLHE41* regulate the development of innate-like B-1a lymphocytes (Kreslavsky et al., 2018). Transgenic mice with dominant-negative Bright/Arid3a had decreased serum IgM, B1 B cells that were functionally deficient in Ig secretion, and poor anti-phosphorylcholine responses which confer protection against *S. pneumoniae* (Nixon et al., 2008) |
| *BHLHE41* | Brain, thyroid | NA | See note from Kreslavasky et al. 2018 above. With knockdown of *Bhlhe41,* B-1a cells were reduced. Mutant B-1a cells had abnormal cell surface and altered B cell receptor repertoire (Kreslavsky et al., 2017). Also *Bhlhe41* and *Bhlhe40* regulate self-renewal of alveolar macrophages (Rauschmeier et al., 2019) |
| *C5AR1* | Whole blood, pituitary, lung, spleen, aorta | Spleen, bone marrow, cerebral cortex, adrenal gland, lung | C5AR modulates TLR4 signaling in macrophages, restraining inflammatory responses to localized infections (Seow et al., 2013). Crosstalk between C5AR and other innate immune receptors increases the proinflammatory response, esp. with pneumococcal induction (van der Maten et al., 2016). C5ar1 deficiency in mice attenuated the IL-1ß response of monocytes and macrophages to LPS challenge (Haggadone et al., 2016), decreased IL-10 production and increased IFN-gamma (Sommerfeld et al., 2021). *C5ar1-*null mice had increased capacity to clear *P. gingivalis* but recruited lower numbers of neutrophils (Maekawa et al., 2014). Similarly *C5ar1-*deficient mice had enhanced clearance of *S. pneumoniae* and reduced severity of acute pneumococcal otitis media after influenza viral infection (Tong et al., 2014) |
| *CPT1B* | Testes, heart, skeletal muscle, brain | Parathyroid gland, testis, heart muscle, skeletal muscle, kidney | CPT1B is a rate-controlling enzyme of long chain fatty acid beta-oxidation pathway in muscle mitochondria. *CPT1B* was upregulated in neonatal sepsis (Misheva et al., 2022). On the other hand, *Cpt1b* was reduced in myocardium after LPS induction (Tzanavari et al., 2016) |
| *CRYBG1* | NA | Parathyroid gland, nasopharynx, bronchus, lung, salivary gland | Possible role in promoting carbohydrate binding activity. *CRYBG1/AIM1* is mutated or methylated in cell lines from extranodal natural killer-T-cell lymphoma (Sako et al., 2014) |
| *FAM227A* | Testes, pituitary, thyroid, fallopian tube, cervix | NA | NA |
| *HEPHL1* | Esophagus, vagina, testes, skin | NA | Enables ferroxidase activity for cellular iron ion homeostasis. Involved as a hub gene in SARS-COV-2 infection (Karami et al., 2021) and upregulated in cutaneous squamous cell carcinoma (Zou et al., 2021) |
| *RAB5A* | Cervical spinal cord, fibroblasts, artery, skin, lung | Thyroid gland, parathyroid gland, adrenal gland, stomach, duodenum | Overexpression of active or dominant-negative mutants of *RAB5A* in macrophages increased phagocytosis of *E. coli* bacteria and cell apoptosis (Frankenberg et al., 2008). *Rab5a* was upregulated in airway epithelial cells during early RSV infection while *Rab5-*knockdown or downregulation reduced lung pathology and disease severity of RSV infection and increased IFN-lambda production (Mo et al., 2021) |
| *RGS22* | Testes, ovaries, fallopian tube, uterus, cervix | NA | Promotes G-protein binding activity; involved in negative regulation of signal transduction. Expressed in several cancers of epithelial origin (Hu et al., 2011) |
| *RTN4* | Fibroblasts, brain tissue, adipose tissue | Cerebellum, caudate, testis, cerebral cortex, hippocampus, soft tissue | Downregulated in epithelia from human and experimental inflammatory bowel disease (Rodriguez-Feo et al., 2015). *RTN4/*NOGO-B overexpression in nasopharyngeal carcinoma increased migration, invasion, and metastatic ability of cancer cells and upregulated p-RhoA, SRF and MRTFA (Wang et al., 2022). RTN4-B deficiency attenuated proinflammatory cytokine production and resulted in impaired TLR9 localization in endolysosomes (Kimura et al., 2015) and impaired transmigration of neutrophils in vascular endothelium (Di Lorenzo et al., 2011) |
| *SPTLC3* | Skin, thyroid, tibial nerve, kidney | Nasopharynx, bronchus, stomach, colon, gallbladder | Upregulated with progressive diabetes in rats (Piccolo et al., 2021), but downregulated in sebum specimens of children with atopic dermatitis (Shima et al., 2022) |
| ^1^All 12 genes were expressed in esophageal mucosa, lungs, cervix, uterus, vagina, minor salivary glands, and skin tissues as a potential surrogate for middle ear mucosa. Tissues with highest expression of candidate genes are listed. | | | |

**REFERENCES for Supplementary Table 3**

Di Lorenzo, A., Manes, T. D., Davalos, A., Wright, P. L., Sessa, W. C. (2011). Endothelial reticulon-4B (Nogo-B) regulates ICAM-1-mediated leukocyte transmigration and acute inflammation. *Blood* 117, 2284-2295. doi: 10.1182/blood-2010-04-281956.

Frankenberg, T., Kirschnek, S., Hacker, H., Hacker, G. (2008). Phagocytosis-induced apoptosis of macrophages is linked to uptake, killing and degradation of bacteria. *Eur. J. Immunol.* 38, 204-215. doi: 10.1002/eji.200737379.

Haggadone, M. D., Grailer, J. J., Fattahi, F., Zetoune, F. S., Ward, P. A. (2016). Bidirectional crosstalk between C5a receptors and the NLRP3 inflammasome in macrophages and monocytes. *Mediators Inflamm.* 2016, 1340156. doi: 10.1155/2016/1340156.

Hu, Y., Xing, J., Wang, L., Huang, M., Guo, X., Chen, L., et al. (2011). RGS22, a novel cancer/testis antigen, inhibits epithelial cell invasion and metastasis. *Clin. Exp. Metastasis* 28, 541-549. doi: 10.1007/s10585-011-9390-z.

Karami, H., Derakhshani, A., Ghasemigol, M., Fereidouni, M., Miri-Moghaddam, E., Baradaran, B., et al. (2021). Weighted gene co-expression network analysis combined with machine learning validation to identify key molecules and hub genes associated with SARS-CoV-2 infection. *J. Clin. Med.* 10, 3567. doi: 10.3390/jcm10163567.

Kimura, T., Endo, S., Inui, M., Saitoh, S. I., Miyake, K., Takai, T. (2015). Endoplasmic protein Nogo-B (RTN4-B) interacts with GRAMD4 and regulates TLR9-mediated innate immune responses. *J. Immunol.* 194, 5426-5436. doi: 10.4049/jimmunol.1402006.

Kreslavsky, T., Vilagos, B., Tagoh, H., Poliakova, D. K., Schwickert, T. A., Wohner, M., et al. (2017). Essential role for the transcription factor Bhlhe41 in regulating the development, self-renewal and BCR repertoire of B-1a cells. *Nat. Immunol.* 18, 442-455. doi: 10.1038/ni.3694.

Kreslavsky, T., Wong, J. B., Fischer, M., Skok, J. A., Busslinger, M. (2018). Control of B-1a cell development by instructive BCR signaling. *Curr. Opin. Immunol.* 51, 24-31. doi: 10.1016/j.coi.2018.01.001.

Maekawa, T., Krauss, J. L., Abe, T., Jotwani, R., Triantafilou, M., Hashim, A., et al. (2014). Porphyromonas gingivalis manipulates complement and TLR signaling to uncouple bacterial clearance from inflammation and promote dysbiosis. *Cell Host Microbe* 15, 768-778. doi: 10.1016/j.chom.2014.05.012.

Misheva, M., Kotzamanis, K., Davies, L. C., Tyrrell, V. J., Rodrigues, P. R. S., Benavides, G. A., et al. (2022). Oxylipin metabolism is controlled by mitochondrial beta-oxidation during bacterial inflammation. *Nat. Commun.* 13, 139. doi: 10.1038/s41467-021-27766-8.

Mo, S., Tang, W., Xie, J., Chen, S., Ren, L., Zang, N., et al. (2021). Respiratory syncytial virus activates Rab5a to suppress IRF1-dependent IFN-gamma production, subverting the antiviral defense of airway epithelial cells. *J. Virol.* 95, e02333-20. doi: 10.1128/JVI.02333-20.

Nixon, J. C., Ferrell, S., Miner, C., Oldham, A. L., Hochgeschwender, U., Webb, C. F. (2008). Transgenic mice expressing dominant-negative bright exhibit defects in B1 B cells. *J. Immunol.* 181, 6913-6922. doi: 10.4049/jimmunol.181.10.6913.

Piccolo, B. D., Graham, J. L., Kang, P., Randolph, C. E., Shankar, K., Yeruva, L., et al. (2021). Progression of diabetes is associated with changes in the ileal transcriptome and ileal-colon morphology in the UC Davis Type 2 Diabetes Mellitus rat. *Physiol. Rep.* 9, e15102. doi: 10.14814/phy2.15102.

Ratliff, M. L., Shankar, M., Guthridge, J. M., James, J. A., Webb, C. F. (2020). TLR engagement induces ARID3A in human blood hematopoietic progenitors and modulates IFN-alpha production. *Cell Immunol.* 357, 104201. doi: 10.1016/j.cellimm.2020.104201.

Rauschmeier, R., Gustafsson, C., Reinhardt, A., Gonzalez, N. A., Tortola, L., Cansever, D., et al. (2019). Bhlhe40 and Bhlhe41 transcription factors regulate alveolar macrophage self-renewal and identity. *EMBO J.* 38, e101233. doi: 10.15252/embj.2018101233.

Rodriguez-Feo, J.A., Puerto, M., Fernandez-Mena, C., Verdejo, C., Lara, J. M., Diaz-Sanchez, M., et al. (2015). A new role for reticulon-4B/NOGO-B in the intestinal epithelial barrier function and inflammatory bowel disease. *Am. J. Physiol. Gastrointest. Liver Physiol.* 308, G981-993. doi: 10.1152/ajpgo.00309.2014.

Sako, N., Dessirier, V., Bagot, M., Bensussan, A., Schmitt, C. (2014). HACE1, a potential tumor suppressor geneon 6q21, is not involved in extranodal natural killer/T-cell lymphoma pathophysiology. *Am. J. Pathol.* 184, 2899-2907. doi: 10.1016/j.ajpath.2014.07.011.

Sari-Ak, D., Torres-Gomez, A., Yazicioglu, Y. F., Christofides, A., Patsoukis, N., Lafuente, E. M., Bioussiotis, V.A. (2022). Structural, biochemical, and functional properties of the Rap1-interacting adaptor molecule (RIAM). *Biomed. J.* 45, 289-298. doi: 10.1016/j.bj.2021.09.005.

Seow, V., Lim, J., Iyer, A., Suen, J. Y., Ariffin, J. K., Hohenhaus, D. M., et al. (2013). Inflammatory responses induced by lipopolysaccharide are amplified in primary human monocytes but suppressed in macrophages by complement protein C5a. *J. Immunol.* 191, 4308-4316. doi: 10.4049/jimmunol.1301355.

Shima, K., Inoue, T., Uehara, Y., Iwamura, M., Fukagawa, S., Kuwano, T., et al. (2022). Non-invasive transcriptomic analysis using mRNAs in skin surface lipids obtained from children with mild-to-moderate atopic dermatitis. *J. Eur. Acad. Dermatol.Venereol.* 36, 1477-1485. doi: 10.1111/jdv.18173.

Sommerfeld, O., Medyukhina, A., Neugebauer, S., Ghait, M., Ulferts, S., Lupp, A., et al. (2021) Targeting complement C5a receptor 1 for the treatment of immunosuppression and sepsis. *Mol. Ther.* 29, 338-346. doi: 10.1016/j.ymthe.2020.09.008.

Sun, H., Lagarrigue, F., Wang, H., Fan, Z., Lopez-Ramirez, M. A., Chang, J. T., Ginsberg, M. H. (2021). Distinct integrin activation pathways for effector and regulatory T cell trafficking and function. *J. Exp. Med.* 218, e20201524. doi: 10.1084/jem.20201524.

Tong, H. H., Lambert, G., Li, Y. X., Thurman, J. M., Stahl, G. L., Douthitt, K., et al. (2014). Deletion of the complement C5a receptor alleviates the severity of acute pneumococcal otitis media following influenza A virus infection in mice. *PLoS One* 9, e95160. doi: 10.1371/journal.pone.0095160.

Tzanavari, T., Varela, A., Theocharis, S., Ninou, E., Kapelouzou, A., Cokkinos, D. V., et al. (2016). Metformin protects against infection-induced myocardial dysfunction. *Metabolism* 65, 1447-1458. doi: 10.1016/j.metabol.2016.06.012.

van der Maten, E., de Bont, C. M., de Groot, R., de Jonge, M. I., Langereis, J. D., van der Flier, M. (2016). Alternative pathway regulation by factor H modulates Streptococcus pneumoniae induced proinflammatory cytokine responses by decreasing C5a receptor crosstalk. *Cytokine* 88, 281-286. doi: 10.1016/j.cyto.2016.09.025.

Wang, J., Zhong, Q., Zhang, H., Liu, S., Li, S., Xia, T., et al. (2022) Nogo-B promotes invasion and metastasis of nasopharyngeal carcinoma via RhoA-SRF-MRTFA pathway. *Cell Death Dis* 13, 76. doi: 10.1038/s41419-022-04518-0.

Zou, D. D., Xu, D., Deng, Y. Y., Wu, W. J., Zhang, J., Huang, L., He, L. (2021). Identification of key genes in cutaneous squamous cell carcinoma: a transcriptome sequencing and bioinformatics profiling study. *Ann. Transl. Med.* 9, 1497. doi: 10.21037/atm-21-3915.
